# A 20-Year Real-World Study of Small Bowel Cancers: Histologic Subtypes, Clinical Features, and Survival Implications

**DOI:** 10.3390/jcm14196962

**Published:** 2025-10-01

**Authors:** Jirapat Wonglhow, Patrapim Sunpaweravong, Chirawadee Sathitruangsak, Arunee Dechaphunkul, Panu Wetwittayakhlang

**Affiliations:** 1 Division of Medical Oncology, Department of Internal Medicine, Faculty of Medicine, Prince of Songkla University, Songkhla 90110, Thailand; jirapat.jw@gmail.com (J.W.); spatrapi@medicine.psu.ac.th (P.S.); sjirawadee@gmail.com (C.S.); dr.arunee@gmail.com (A.D.); 2 Gastroenterology and Hepatology Unit, Division of Internal Medicine, Faculty of Medicine, Prince of Songkla University, Songkhla 90110, Thailand

**Keywords:** small bowel cancer, histology, adenocarcinoma, neuroendocrine tumor, GIST, prognosis, prognostic factors, prevalence

## Abstract

**Background:** Small-bowel cancers (SBCs) are rare, histologically diverse malignancies with limited data from Asian populations. This study aimed to describe histological subtype distribution, clinical features, survival outcomes, and prognostic factors in SBCs over a 20-year period. **Methods:** We retrospectively reviewed patients diagnosed with SBC at a tertiary referral center in Southern Thailand (2005–2024). Clinical, pathological, and radiological data were analyzed by histologic subtype. **Results:** A total of 158 patients were included: adenocarcinoma (81.0%), gastrointestinal stromal tumor (GIST, 5.7%), well-differentiated neuroendocrine tumor (NET, 5.7%), other sarcomas (5.1%), and poorly differentiated neuroendocrine carcinoma (NEC, 2.5%). Adenocarcinoma predominantly affected older patients and frequently presented with advanced-stage disease and poor performance status, whereas NET and NEC occurred in younger patients typically at early NET and metastatic NEC stages. Median overall survival (OS) varied by subtype: adenocarcinoma (8.3 months), GIST (63.6 months), NEC (8.9 months), NET (not reached), and other sarcomas (9.8 months). Five-year OS rates were 14.0%, 55.6%, 0%, 88.9%, and 18.8%, respectively. Eastern Cooperative Oncology Group performance status ≥2, duodenal location, and metastatic disease were independently associated with worse OS. **Conclusions:** SBCs display distinct clinical and prognostic profiles by subtype. Overall prognosis remained poor, underscoring the need for earlier detection and subtype-specific management.

## 1. Introduction

Small bowel cancers (SBCs) are a rare and heterogeneous group of gastrointestinal malignancies accounting for approximately 3–5% of all gastrointestinal cancers worldwide [1]. Although the small intestine comprises >70% of the length and 90% of the absorptive surface area of the gastrointestinal tract, malignancies arising from this segment are uncommon [2]. Among them, the duodenum accounts for the majority of SBCs (55–88%), followed by the jejunum (11–25%) and ileum (7–17%) [3]. The most frequently reported histological subtypes of SBCs include adenocarcinoma, neuroendocrine neoplasms, which are further classified into well-differentiated neuroendocrine tumors (NETs) and poorly differentiated neuroendocrine carcinomas (NECs) according to the WHO 5th edition, gastrointestinal stromal tumors (GISTs), and other sarcomas, each with a distinct biological behavior, tumor location, and clinical course [4,5,6].

Adenocarcinoma has historically been the most common subtype in Western registries [7], often presenting in the duodenum and associated with late-stage diagnosis and poor prognosis [8]. In contrast, NETs are increasingly being recognized in the ileum and jejunum and are often diagnosed at earlier stages with relatively favorable outcomes [9]. GISTs arising from the interstitial cells of Cajal are more commonly detected in the jejunum or ileum and can exhibit indolent or aggressive behavior depending on their size and mitotic index [10]. Sarcomas are the rarest subtype, with diverse prognoses [11].

The rarity and histological diversity of SBCs have resulted in limited prospective studies and a paucity of population-based data, particularly in Asian populations, where genetic and environmental factors may influence cancer epidemiology. The existing literature has largely focused on individual subtypes or specific tumor locations, with few studies comprehensively evaluating the subtype distribution, clinical presentation, staging at diagnosis, treatment patterns, and survival outcomes across the entire spectrum of small bowel malignancies [12,13].

Given the diagnostic challenges and clinical heterogeneity associated with SBCs, a detailed understanding of their epidemiological and clinical characteristics is essential for early diagnosis and optimal management. This study aimed to fill this knowledge gap by providing a comprehensive retrospective analysis of the histologic subtypes, clinicopathological and radiological correlations, and survival outcomes of SBCs in a real-world cohort from a tertiary center in Southern Thailand.

## 2. Materials and Methods

### 2.1. Study Design and Setting

This was a retrospective study of patients diagnosed with SBC at the Songklanagarind Hospital, Prince of Songkla University, between January 2005 and December 2024.

### 2.2. Patient Selection

Patients were first identified by searching the hospital electronic medical record system for all encounters coded with ICD-10 diagnosis C17.x (malignant neoplasm of small intestine). Eligible patients were identified based on the following criteria: (1) age of 18 years or older at diagnosis and (2) histologically confirmed malignancy of the small intestine (duodenum, jejunum, or ileum). Patients were excluded if they had (1) non-small bowel primary cancers that metastasized to the small bowel, (2) primary periampullary or ampullary cancer, (3) benign small bowel tumors or premalignant lesions without evidence of malignancy, (4) small bowel involvement from lymphomas, or (5) no histopathological diagnosis. The case selection process is summarized in the STROBE flow diagram (Figure S1).

### 2.3. Data Collection

Patient demographic and clinical data were systematically collected from electronic hospital records. The key variables included age at diagnosis, sex, body mass index, Eastern Cooperative Oncology Group (ECOG) performance status, histological subtype, anatomical site of the primary tumor, pathological TNM classification, treatment history, and follow-up information. Radiologic data were obtained from contrast-enhanced computed tomography (CT) reports, and histopathological information was extracted from the pathology reports in the electronic medical record. No additional centralized radiology and pathology review was performed as part of this retrospective study.

The study protocol was reviewed and approved by the Ethics Committee of the Research Centre of the Faculty of Medicine, Prince of Songkla University (REC.68304141). The requirement for written informed consent was waived due to the retrospective nature of the study. All data were anonymized to ensure patient confidentiality.

### 2.4. Outcome Measures

The primary aim of this study was to determine the prevalence of various histologic subtypes of SBC. The secondary objectives were to examine the association between histologic subtype and both clinicopathological and radiological characteristics, and to explore survival outcomes and potential prognostic factors for overall survival (OS) of SBCs across histologic subtypes. OS was defined as the time from histologically confirmed diagnosis to death from any cause.

### 2.5. Statistical Analysis

Descriptive statistics were used to summarize the baseline characteristics. Continuous variables are reported as median with interquartile range (IQR) or mean with standard deviation (SD), depending on the data distribution. Categorical variables are presented as counts and percentages. Chi-square or Fisher’s exact tests were used to analyze the association between histology and clinical, pathological, and radiological characteristics. Survival outcomes were estimated using the Kaplan–Meier method, and comparisons between groups were performed using the log-rank test. Univariate Cox proportional hazards regression was used to assess the factors associated with OS, and potentially significant variables were then included in multivariate Cox regression analysis to identify independent prognostic factors. The proportional hazards assumption was assessed using Schoenfeld residuals. Model stability was evaluated by calculating events-per-variable (EPV) for the final multivariable model, with an EPV threshold of ≥10 considered acceptable. All statistical analyses were performed using the R software version 4.3.1 (R Foundation for Statistical Computing, Vienna, Austria). Two-sided *p*-values were reported, with values less than 0.05 considered indicative of statistical significance.

## 3. Results

### 3.1. Prevalence of Small Bowel Cancer Across Histologic Subtypes

**Figure 1:**
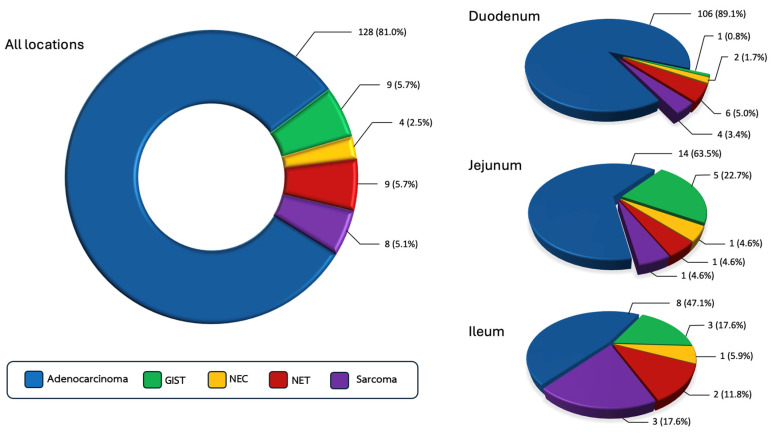
Prevalence of small bowel cancer stratified by histologic subtypes and primary tumor location.

In total, 158 patients with SBC were included in this study. Among these, 128 (81.0%) had adenocarcinoma. Both GIST and well-differentiated NETs were identified in 9 patients (5.7%), while 8 patients (5.1%) had other sarcomas (6 leiomyosarcomas, 1 clear cell sarcoma, 1 unspecified sarcoma) and 4 patients (2.5%) had poorly differentiated NEC. When stratified by tumor location (Figure 1), adenocarcinoma (89.1%) was the most common subtype in the duodenum, followed by NET (5.0%), other sarcomas (3.4%), NEC (1.7%), and GIST (0.8%). In the jejunum, adenocarcinoma (63.5%) remained the most frequent, followed by GIST (22.7%), with other sarcomas (4.6%), NET (4.6%), and NEC (4.6%) occurring in equal proportions. In the ileum, adenocarcinoma (47.1%) was also the most prevalent, followed by other sarcomas (17.6%), GIST (17.6%), NET (11.8%), and NEC (5.9%).

### 3.2. Demographics

The majority of patients were male (55.7%), with a similar sex distribution across the subtypes (Table 1). The median age at diagnosis was 61.2 years (range, 23.4–86.7). Patients with adenocarcinoma had the highest mean age (62.2 years), and 31.2% were aged ≥70. In contrast, no patients with NEC (mean age 42.2 years) or other sarcomas (mean age 49.7 years) were aged ≥70, and mean ages were lower for those with GIST (50.3 years) and NET (57.3 years), indicating a younger age at diagnosis for these subtypes compared to adenocarcinoma. Additionally, patients with adenocarcinoma had a higher prevalence of poor performance status (ECOG ≥ 2) (42.2%). All patients with NET had an ECOG score of 0–1.

### 3.3. Tumor Location

**Figure 2:**
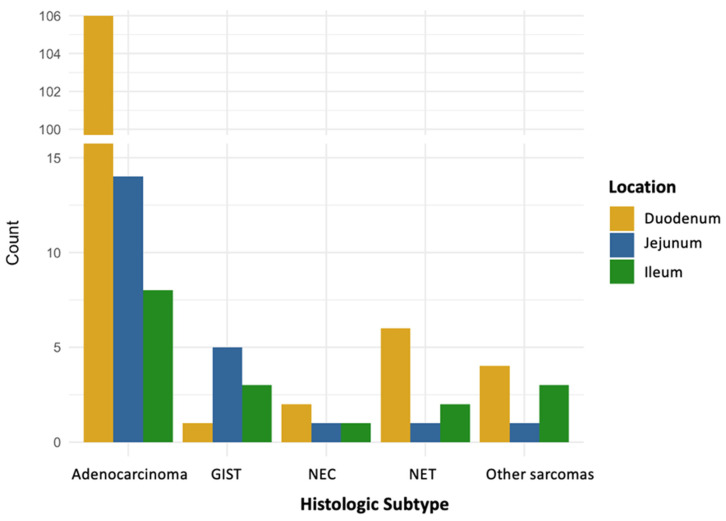
Tumor location distribution by histologic subtype. GIST, gastrointestinal stromal tumor; NEC, neuroendocrine carcinoma; NET, neuroendocrine tumor.

The duodenum was the most common tumor site (75.3%), particularly in adenocarcinomas (82.8%) and nearly all subtypes (Figure 2), except for GISTs, which were most frequently located in the jejunum (55.6%) (Table 1).

### 3.4. Clinical Presentation

The most frequently presenting symptoms were abdominal pain (34.2%) and gastric outlet obstruction (31.0%), followed by jaundice (19.0%) and weight loss (15.8%) (Table 1). Importantly, palpable abdominal masses were reported only in mesenchymal tumors, including GIST (22.2%) and other sarcomas (37.5%), and were absent in all epithelial tumors (adenocarcinomas, NET, and NEC). Jaundice was reported mainly in patients with adenocarcinomas (22.7%), which is consistent with its predominant duodenal location. Bowel perforation was rare (3.8%) and was observed only in adenocarcinomas.

### 3.5. Radiologic Characteristics

Most tumors across the subtypes presented as a detectable mass on imaging. However, 36.7% of the adenocarcinomas appeared with circumferential bowel wall thickening, a feature that is rarely observed in other subtypes. Tumor size ≥5 cm was more common in NEC (66.7%), GIST (87.5%), and other sarcomas (62.5%) compared to adenocarcinoma and NET, which typically presented with smaller lesions.

### 3.6. Staging and Metastatic Pattern

Half of the adenocarcinomas were metastatic at the time of diagnosis. All patients with NEC presented with metastatic disease, whereas all patients with NET cases were diagnosed at an early stage. GIST and other sarcomas were predominantly localized at the time of diagnosis (Table 1). Common metastatic sites for adenocarcinomas and NEC included the liver, peritoneum, and lymph nodes. Lung metastasis occurred most frequently in patients with other sarcomas. Notably, lymph node involvement was not observed in the GIST or other sarcomas.

Among 64 patients with non-metastatic adenocarcinoma, 52 (81.3%) underwent surgical resection. Of those resected patients, the majority had locally advanced disease (pathological T4 in 92.3%, and pathological T3 in 7.7%). Nodal involvement was observed in 26.9% (pathological N1 in 19.2%, and pathological N2 in 7.7%), and four patients (7.7%) had microscopically positive margins (R1 resection).

### 3.7. Laboratory Findings and Tumor Markers

Anemia (hemoglobin < 10 g/dL) and hypoalbuminemia (albumin < 3.5 g/dL) were most common in patients with adenocarcinoma and NEC. Carcinoembryonic antigen (CEA) and carbohydrate antigen 19-9 (CA19-9) levels were tested almost exclusively in adenocarcinoma and were elevated in 44% and 67% of the tested patients, respectively. These markers were not elevated in the other subtypes (Table 1).

### 3.8. Survival Outcomes

**Figure 3:**
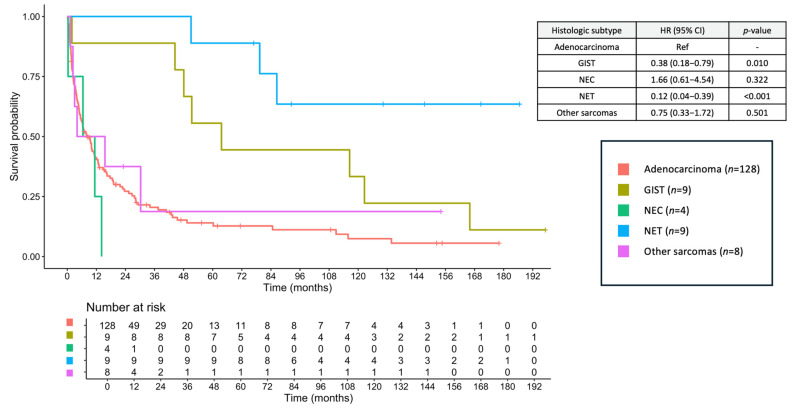
Overall survival of small bowel cancers across histologic subtypes. GIST, gastrointestinal stromal tumor; NEC, neuroendocrine carcinoma; NET, neuroendocrine tumor.

The median follow-up duration was 9.79 months (range, 0.16–197.24 months). The median OS for the entire cohort was 6.87 months (95% confidence interval [CI], 6.87–15.60), with a 2-year OS of 35.1% and a 5-year OS of 21.2%. When stratified by histologic subtype, the median OS was 8.28 months (95% CI, 5.45–12.60) for adenocarcinoma, 63.60 months (95% CI, 48.01–NA) for GIST, 8.90 months (95% CI, 0.33–NA) for NEC, not reached (NR) (95% CI, 86.43–NA) for NET, and 9.79 months (95% CI, 3.02–NA) for other sarcomas (Figure 3). The corresponding 2-year OS rates were 20.5% (adenocarcinoma), 88.9% (GIST), 0% (NEC), 100% (NET), and 37.5% (other sarcomas), and the corresponding 5-year OS rates were 14.0, 55.6, 0, 88.9, and 18.8%, respectively.

**Figure 4:**
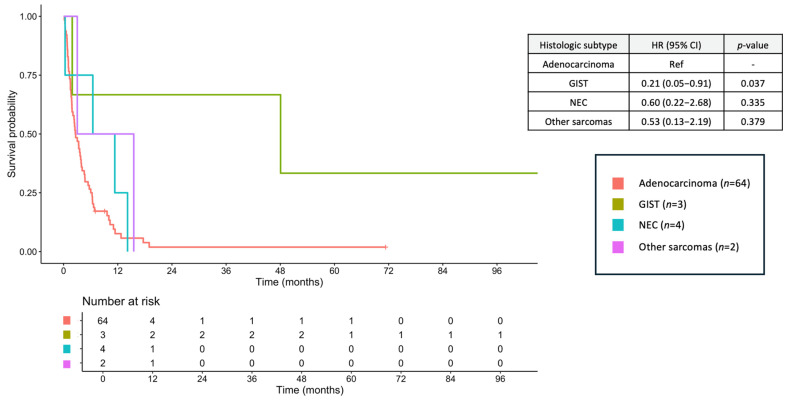
Overall survival of metastatic stage small bowel cancers across histologic subtypes. GIST, gastrointestinal stromal tumor; NEC, neuroendocrine carcinoma.

Among patients with metastatic disease, the median OS was 3.02 months (95% CI, 2.30–4.57), with a 2-year OS of 4.7% and a 5-year OS of 3.1%. Within this subgroup, median OS was 2.63 months (95% CI, 1.87–3.94) for adenocarcinoma, 48.06 months (95% CI, 1.91–NA) for GIST, 8.90 months (95% CI, 0.33–NA) for NEC, and 9.28 months (95% CI, 3.02–NA) for other sarcomas (Figure 4). Palliative chemotherapy was administered to 19 patients (29.7%) with synchronous metastatic adenocarcinoma (FOLFOX, *n* = 8; CAPOX, *n* = 6; cisplatin/5-FU, *n* = 1; single-agent 5-FU, *n* = 4). Three patients with NEC (75.0%) received platinum/etoposide, all two patients with other sarcomas received palliative chemotherapy (doxorubicin/cyclophosphamide, *n* = 1; single-agent doxorubicin, *n* = 1), and two patients with metastatic GIST (66.7%) received imatinib.

**Figure 5:**
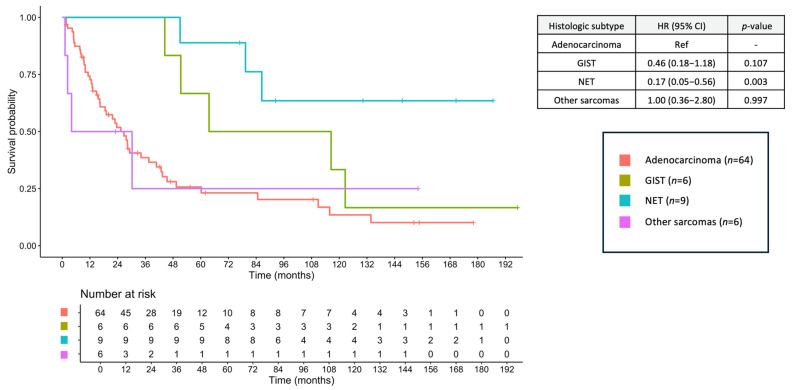
Overall survival of non-metastatic stage small bowel cancers across histologic subtypes. GIST, gastrointestinal stromal tumor; NEC, neuroendocrine carcinoma.

In the non-metastatic cohort, the median OS was 34.10 months (95% CI, 25.40–51.30), with a 2-year OS of 60.7% and a 5-year OS of 36.5%. Stratified by subtype, median OS was 25.39 months (95% CI, 16.43–40.80) for adenocarcinoma, 90.00 months (95% CI, 51.35–NA) for GIST, NR (95% CI, 86.43–NA) for NET, and 17.1 months (95% CI, 2.33–NA) for other sarcomas (Figure 5). Among 64 resected adenocarcinomas, 29 (45.3%) received adjuvant chemotherapy (single-agent 5-FU, *n* = 14; FOLFOX, *n* = 7; CAPOX, *n* = 5; capecitabine monotherapy, *n* = 3). One patient with GIST (16.7%) received adjuvant imatinib, and one patient with other sarcoma (16.7%) received adjuvant doxorubicin/cyclophosphamide.

### 3.9. Prognostic Factors for OS

Multivariate Cox proportional hazard regression analysis identified poor ECOG performance status, a primary tumor located in the duodenum, and the presence of metastatic disease at diagnosis as independent factors for worse OS (Table 2). The proportional hazards assumption was not violated (global Schoenfeld residual test *p* = 0.36). The final multivariable model included 150 patients with 126 events, yielding an EPV of 10.5, which supports model stability.

## 4. Discussion

This 20-year retrospective study provides a comprehensive overview of the clinical, pathological, and radiological characteristics, as well as the survival outcomes of SBCs in a real-world cohort from Southern Thailand. Our findings reaffirm the heterogeneity of SBCs and highlight the substantial differences in patient demographics, tumor biology, and prognosis across histological subtypes.

Adenocarcinoma accounted for the majority of cases in our cohort, which is consistent with studies from Western countries [3,7]. However, the prevalence in our population (81.0%) was notably higher than that reported in previous studies (typically 30–50%). Patients with small bowel adenocarcinoma (SBA) are generally older and more likely to present with poor performance status and advanced-stage disease, underscoring its aggressive clinical course [4,14]. Abdominal pain is a common symptom across subtypes; however, the presence of jaundice is highly specific to adenocarcinoma, consistent with its predominant duodenal location [15]. Our findings also confirmed that SBAs were most often located in the duodenum and frequently presented as masses <5 cm in size. Interestingly, one-third of the patients with SBA presented with circumferential bowel wall thickening, a radiologic feature rarely observed in other subtypes. These findings suggest that SBA should be suspected, although not exclusively, when elderly patients present with jaundice, duodenal masses, or focal bowel wall thickening <5 cm, particularly in the setting of metastatic disease.

Survival outcomes for SBA were poor, with a 5-year OS of only 14.0%, which was lower than that reported in other studies [3,16]. Only 60% of the patients with metastatic adenocarcinoma received palliative chemotherapy, and median OS in the metastatic vs. non-metastatic cases was markedly different (2.63 vs. 25.39 months). SBA also has a poorer prognosis than large bowel adenocarcinoma, even though the same treatment approach was applied [17]. These results underscore the need for improved early detection and expanded access to timely and effective treatments in this population.

Neuroendocrine neoplasms, comprising well-differentiated NETs and poorly differentiated NECs [18], represented a small proportion of cases in our study (5.7% and 2.5%, respectively), lower than that previously reported (approximately 35%) [3,7,19], which contrasts with some studies revealing that NET was the most common subtype [2,20]. Despite their similarity, NETs and NECs have distinct biologies and outcomes. Previous studies have reported favorable outcomes only for NETs [9,19]; however, NECs should be evaluated separately. In this study, all NET cases were diagnosed at an early stage, with no metastatic disease at presentation, and showed excellent long-term survival (5-year OS, 88.9%). In contrast, all patients with NEC presented with metastatic disease and had a median OS of 8.90 months. These findings highlight the importance of accurately distinguishing NEC from other subtypes to ensure prompt systemic therapy. A limitation of this study is the incomplete documentation of Ki-67 index, mitotic count, and functionality in NET/NEC cases, precluding grading of NETs into G1–G3. Therefore, all cases were classified as well-differentiated NET and poorly differentiated NEC based on morphology, consistent with 5th edition WHO criteria [6].

Both NET and NEC were diagnosed in younger patients than adenocarcinomas, with patients with NEC showing the lowest mean age. While the clinical presentations are similar to those of SBA, jaundice is less common, likely due to the lower prevalence of duodenal primaries in these subtypes [7,21]. Tumor markers such as CEA and CA19-9 are potential markers that are elevated only in adenocarcinoma. However, only 44% and 67% of the tested cases showed abnormal values for adenocarcinoma. Hence, positive tumor markers could guide the differential diagnosis of adenocarcinoma, but it could not be distinguished if they were negative. Nonetheless, this should be interpreted with caution because of the presence of missing data in this study (approximately 45% for CEA and 60% for CA19-9) which may limit their reliability in the differential diagnosis. Tumor markers should be further evaluated in the context of SBC and their prognostic roles.

Sarcomas were the second most common SBC subtype in our study, an uncommon finding compared to other series in which NET were typically more prevalent [7]. GIST was the most common sarcoma subtype and was analyzed separately due to its distinct clinical behavior and favorable response to the targeted imatinib therapy [3,22,23]. Our study found that both GIST and other sarcomas typically present as large masses, and the abdominal mass is an exclusive feature of these mesenchymal tumors. Unlike most subtypes, GISTs are predominantly located in the jejunum and ileum [7]. Importantly, lymph node involvement was not observed in the sarcomas, consistent with their tendency to metastasize via the hematogenous route, particularly to the lungs [24]. GIST demonstrated a favorable prognosis, with a 5-year OS of 55.6%, even among some patients with metastatic disease, likely reflecting the efficacy of tyrosine kinase inhibitors such as imatinib [25]. In contrast, other sarcomas have shown outcomes similar to those of SBA. Taken together, sarcomas, particularly GIST, should be suspected when patients present with an abdominal mass, jejunal or ileal location, and no evidence of lymph node involvement.

An important insight from our study was the association between tumor location and prognosis. Overall, duodenal tumors were the most common, except for GIST, which predominantly occurred in the jejunum. The multivariate analysis revealed that tumors located in the jejunum or ileum were associated with significantly better survival than those located in the duodenum. This may reflect earlier detection, less invasive behavior, or higher resectability [15]. Other independent prognostic factors included the ECOG performance status and metastatic stage. The histologic subtype was also a strong determinant of outcome, with NET and GIST showing significantly better survival than adenocarcinoma [3,16,19]. Of note, tumor markers such as CEA and CA19-9 were not analyzed in the survival models because of substantial missing data, which may have limited our ability to evaluate their prognostic value. Further studies should explore their prognostic value in the context of SBC. Although some factors were not significant in our cohort, previous studies have demonstrated that hypoalbuminemia and bowel obstruction are associated with poorer survival outcomes [26].

Our study has several strengths, including being one of the largest single-institution cohorts of SBCs in Southeast Asia, as well as long-term follow-up and a comprehensive evaluation of histologic subtypes. However, this study had several limitations. The retrospective nature of the study introduced a potential selection bias, and certain parameters, such as molecular profiling and mitotic index (particularly in GISTs), were not consistently available. Radiologic findings were abstracted from official CT reports, and no centralized re-review of imaging was performed. Therefore, inter-reader variability in the interpretation of mass versus circumferential wall thickening may exist. Additionally, treatment data were limited, and differences in therapeutic approaches may have affected survival outcomes. Because of the small number of patients in most histologic subtypes and treatment subgroups, formal subgroup or sensitivity analyses were not performed, as these would be statistically underpowered and potentially misleading. Information on cause of death was frequently unavailable, precluding cancer-specific survival analysis, which may have provided further insights into disease-specific outcomes. Another limitation of this study is that management of SBC has evolved over the past two decades, with wider use of targeted therapy, improved peri-operative care, and better imaging. These changes may have influenced stage distribution, treatment, and outcomes, but the relatively small sample size in each time period limited our ability to perform meaningful stratified analyses. Larger, multi-center cohorts would be better-suited to explore temporal trends.

Despite these limitations, our study provided valuable real-world insights into the clinicopathological spectrum of SBCs. This emphasizes the prognostic relevance of histologic subtype, disease stage, and tumor location. These findings suggest the need for subtype-specific diagnostic strategies and treatment algorithms. Future prospective multicenter studies with standardized molecular profiling are warranted to validate these observations and to explore novel biomarker-driven therapies for this rare and heterogeneous disease group.

## 5. Conclusions

SBCs represent a rare but clinically heterogeneous group of malignancies with distinct characteristics across histological subtypes. Our 20-year real-world analysis highlighted that adenocarcinoma is the predominant subtype and is associated with older age, advanced-stage presentation, and poor prognosis. In contrast, NET and GIST demonstrate a more favorable tumor biology and outcomes, whereas NECs have a particularly poor prognosis. Tumor location, performance status, and metastatic stage are key prognostic determinants. Notably, the OS of patients with SBC in our Thai cohort appeared shorter than that reported in Western series, which may partly reflect differences in primary site distribution, stage at presentation, and access to systemic therapy. These findings underscore the need to improve early detection, diagnostic accuracy, and access to optimal treatment strategies to enhance outcomes in patients with SBC.

## Figures and Tables

**Table 1:** Baseline characteristics.

	Adenocarcinoma (*n* = 128)	GIST (*n* = 9)	NEC (*n* = 4)	NET (*n* = 9)	Other Sarcomas (*n* = 8)
Sex, *n* (%) Female Male	59 (46.1) 69 (53.9)	3 (33.3) 6 (66.7)	2 (50.0) 2 (50.0)	3 (33.3) 6 (66.7)	3 (37.5) 5 (62.5)
Age, years (SD) * Age ≥ 70 years, *n* (%)	62.2 (12.6) 40 (31.2)	50.3 (15.4) 1 (11.1)	42.2 (15.3) 0 (0)	57.3 (16.6) 2 (22.2)	49.7 (13.4) 0 (0)
BMI, *n* (%) <18.5 kg/m^2^ 18.5–22.9 kg/m^2^ ≥23.0 kg/m^2^	51 (39.8) 45 (35.2) 32 (25.0)	4 (44.4) 3 (33.3) 2 (22.2)	0 (0) 2 (50.0) 2 (50.0)	2 (22.2) 4 (44.4) 3 (33.3)	2 (25.0) 4 (50.0) 2 (25.0)
ECOG PS, *n* (%) * 0–1 ≥2	74 (57.8) 54 (42.2)	8 (88.9) 1 (11.1)	3 (75.0) 1 (25.0)	9 (100) 0 (0)	6 (75.0) 2 (25.0)
Tumor location, *n* (%) * Duodenum Jejunum Ileum	106 (82.8) 14 (10.9) 8 (6.2)	1 (11.1) 5 (55.6) 3 (33.3)	2 (50.0) 1 (25.0) 1 (25.0)	6 (66.7) 1 (11.1) 2 (22.2)	4 (50.0) 1 (12.5) 3 (37.5)
Clinical presentation, *n* (%) # Bowel obstruction Bowel perforation Abdominal pain Abdominal distension Abdominal mass * Significant weight loss Melena Chronic diarrhea Jaundice Anemia Anorexia	44 (34.4) 4 (3.1) 42 (32.8) 8 (6.2) 0 (0) 23 (18.0) 11 (8.6) 3 (2.3) 29 (22.7) 4 (3.1) 10 (7.8)	0 (0) 0 (0) 4 (44.4) 1 (11.1) 2 (22.2) 0 (0) 1 (11.1) 0 (0) 0 (0) 1 (11.1) 0 (0)	1 (25.0) 0 (0) 2 (50.0) 0 (0) 0 (0) 2 (50.0) 0 (0) 0 (0) 0 (0) 0 (0) 0 (0)	2 (22.2) 0 (0) 4 (44.4) 0 (0) 0 (0) 0 (0) 2 (22.2) 0 (0) 1 (11.1) 0 (0) 0 (0)	2 (25.0) 2 (25.0) 2 (25.0) 0 (0) 3 (37.5) 0 (0) 0 (0) 0 (0) 0 (0) 0 (0) 1 (12.5)
Lesion from imaging, *n* (%) Mass Bowel wall thickening Undetected lesion	73 (57.0) 47 (36.7) 8 (6.2)	8 (88.9) 1 (11.1) 0 (0)	3 (75.0) 1 (25.0) 0 (0)	6 (66.7) 1 (11.1) 2 (22.2)	7 (87.5) 1 (12.5) 0 (0)
Tumor size Largest diameter of mass, cm (IQR) <5 cm, *n* (%) ≥5 cm, *n* (%) NA, *n* (%)	(*n* = 73) 4.4 (3.2, 5.6) 34 (46.6) 20 (27.4) 19 (26.0)	(*n* = 8) 7.5 (7.0, 13.0) 0 (0) 7 (87.5) 1 (12.5)	(*n* = 3) 11.9 (11.5, 12.4) 0 (0) 2 (66.7) 1 (33.3)	(*n* = 6) 3.8 (2.8, 6.2) 3 (50.0) 1 (16.7) 2 (33.3)	(*n* = 8) 8.0 (5.2, 9.8) 2 (25.0) 5 (62.5) 1 (12.5)
Stage, *n* (%) * Non-metastatic disease Metastatic disease	64 (50.0) 64 (50.0)	6 (66.7) 3 (33.3)	0 (0) 4 (100)	9 (100) 0 (0)	6 (75.0) 2 (25.0)
Organ metastasis, *n* (%) Peritoneum Lymph node Lung Liver Bone Pleura Adrenal Ovary Brain	26 (20.3) 30 (23.4) 8 (6.2) 25 (19.5) 0 (0) 2 (1.6) 1 (0.8) 4 (3.1) 0 (0)	1 (11.1) 0 (0) 0 (0) 2 (22.2) 0 (0) 0 (0) 1 (11.1) 0 (0) 0 (0)	1 (25.0) 2 (50.0) 0 (0) 2 (50.0) 0 (0) 0 (0) 0 (0) 0 (0) 0 (0)	- - - - - - - - -	1 (12.5) 0 (0) 2 (25.0) 1 (12.5) 1 (12.5) 0 (0) 0 (0) 0 (0) 1 (12.5)
Number of metastases, *n* (%) 1 ≥2	38 (29.7) 26 (20.3)	2 (22.2) 1 (11.1)	3 (75.0) 1 (25.0)	- -	0 (0) 2 (25.0)
Tumor differentiation, *n* (%) Well Moderately Poorly	43 (33.6) 37 (28.9) 48 (37.5)	- - -	0 (0) 0 (0) 4 (100)	9 (100) 0 (0) 0 (0)	- - -
CEA level, *n* (%) <5 ng/mL ≥5 ng/mL	(*n* = 77) 43 (55.8) 34 (44.2)	(*n* = 4) 4 (100) 0 (0)	(*n* = 3) 3 (100) 0 (0)	(*n* = 1) 1 (100) 0 (0)	(*n* = 2) 2 (100) 0 (0)
CA19-9 level, *n* (%) <37 U/mL ≥37 U/mL	(*n* = 57) 32 (56.1) 25 (43.9)	(*n* = 3) 3 (100) 0 (0)	(*n* = 1) 1 (100) 0 (0)	(*n* = 1) 1 (100) 0 (0)	(*n* = 1) 1 (100) 0 (0)
Hemoglobin, *n* (%) <10 g/dL ≥10 g/dL	(*n* = 126) 63 (50.0) 63 (50.0)	(*n* = 7) 5 (71.4) 2 (28.6)	(*n* = 3) 2 (66.7) 1 (33.3)	(*n* = 6) 2 (33.3) 4 (66.7)	(*n* = 8) 3 (37.5) 5 (62.5)
Albumin, *n* (%) <3.5 g/dL ≥3.5 g/dL	(*n* = 126) 61 (48.4) 65 (51.6)	(*n* = 7) 1 (14.3) 6 (85.7)	(*n* = 3) 2 (66.7) 1 (33.3)	(*n* = 6) 3 (50.0) 3 (50.0)	(*n* = 8) 1 (12.5) 7 (87.5)
Treatment, *n* (%) Primary tumor resection Adjuvant systemic treatment Palliative systemic treatment	59/128 (46.1) 29/64 (45.3) 19/64 (29.7)	7/9 (77.8) 1/6 (16.7) 2/3 (66.7	1/4 (25.0) 0 (0) 3/4 (75.0)	9/9 (100) 0 (0) 0 (0)	5/8 (62.5) 1/6 (16.7) 2/2 (100)

* statistically significant difference across histologic subtypes. # can have more than one answer. GIST, gastrointestinal stromal tumor; NEC, neuroendocrine carcinoma; NET, neuroendocrine tumor; SD, standard deviation; IQR, interquartile range; BMI, body mass index; ECOG, Eastern Cooperative Oncology Group; PS, performance status; NA, not available; CEA, carcinoembryonic antigen; CA19-9, cancer antigen 19-9.

**Table 2:** Prognostic Factors for OS.

	Univariate		Multivariate	
	HR (95% CI)	*p* Value	HR (95% CI)	*p* Value
Age ≥ 70 years	1.62 (1.11–2.36)	0.013	1.23 (0.81–1.88)	0.331
Male vs. Female	0.94 (0.67–1.33)	0.739	-	-
BMI <18.5 vs. ≥18.5 kg/m^2^	1.32 (0.92–1.88)	0.131	-	-
ECOG PS 0–1 vs. 2–3	0.17 (0.11–0.25)	<0.001	0.28 (0.17–0.44)	<0.001
Hemoglobin ≥ 10 g/dL	0.72 (0.51–1.02)	0.066	-	-
Albumin ≥ 3.5 g/dL	0.57 (0.40–0.81)	0.002	0.69 (0.46–1.02)	0.064
Location: Duodenum Jejunum Ileum	Ref 0.41 (0.23–0.73) 0.48 (0.27–0.85)	Ref 0.003 0.011	Ref 0.45 (0.23–0.89) 0.46 (0.23–0.90)	Ref 0.021 0.024
Metastatic stage	5.10 (3.50–7.45)	<0.001	5.27 (3.33–8.34)	<0.001
Bowel obstruction	1.17 (0.81–1.71)	0.406	-	-
Bowel perforation	2.04 (0.89–4.67)	0.090	-	-
Jaundice	1.32 (0.86–2.03)	0.211	-	-
Lesion Mass Bowel wall thickening Undetected	Ref 1.47 (1.20–2.14) 0.80 (0.39–1.65)	Ref 0.048 0.545	Ref 1.31 (0.88–1.97) 0.60 (0.28–1.29)	Ref 0.186 0.187
Size ≥5 cm	1.09 (0.72–1.65)	0.68	-	-
Histology Adenocarcinoma GIST NEC NET Other sarcomas	Ref 0.38 (0.18–0.79) 1.66 (0.61–4.54) 0.12 (0.04–0.39) 0.75 (0.33–1.72)	Ref 0.010 0.322 <0.001 0.501	Ref 0.47 (0.18–1.27) 1.72 (0.50–5.96) 0.35 (0.11–1.18) 1.79 (0.72–4.44)	Ref 0.136 0.394 0.090 0.207

BMI, body mass index; ECOG, Eastern Cooperative Oncology Group; PS, performance status; GIST, gastrointestinal stromal tumor; NEC, neuroendocrine carcinoma; NET, neuroendocrine tumor.

## Data Availability

The datasets used and/or analyzed in the current study are available from the corresponding author upon reasonable request.

## Supplementary Materials

The following supporting information can be downloaded at: https://www.mdpi.com/article/10.3390/jcm14196962/s1, Figure S1 STROBE flow diagram.
