# Supplementary figures and images for: A 20-Year Real-World Study of Small Bowel Cancers: Histologic Subtypes, Clinical Features, and Survival Implications
Source: J Clin Med. 2025 Oct 1;14(19):6962. doi: 10.3390/jcm14196962 (PMC12525336; doi:10.3390/jcm14196962)

## Supplementary data

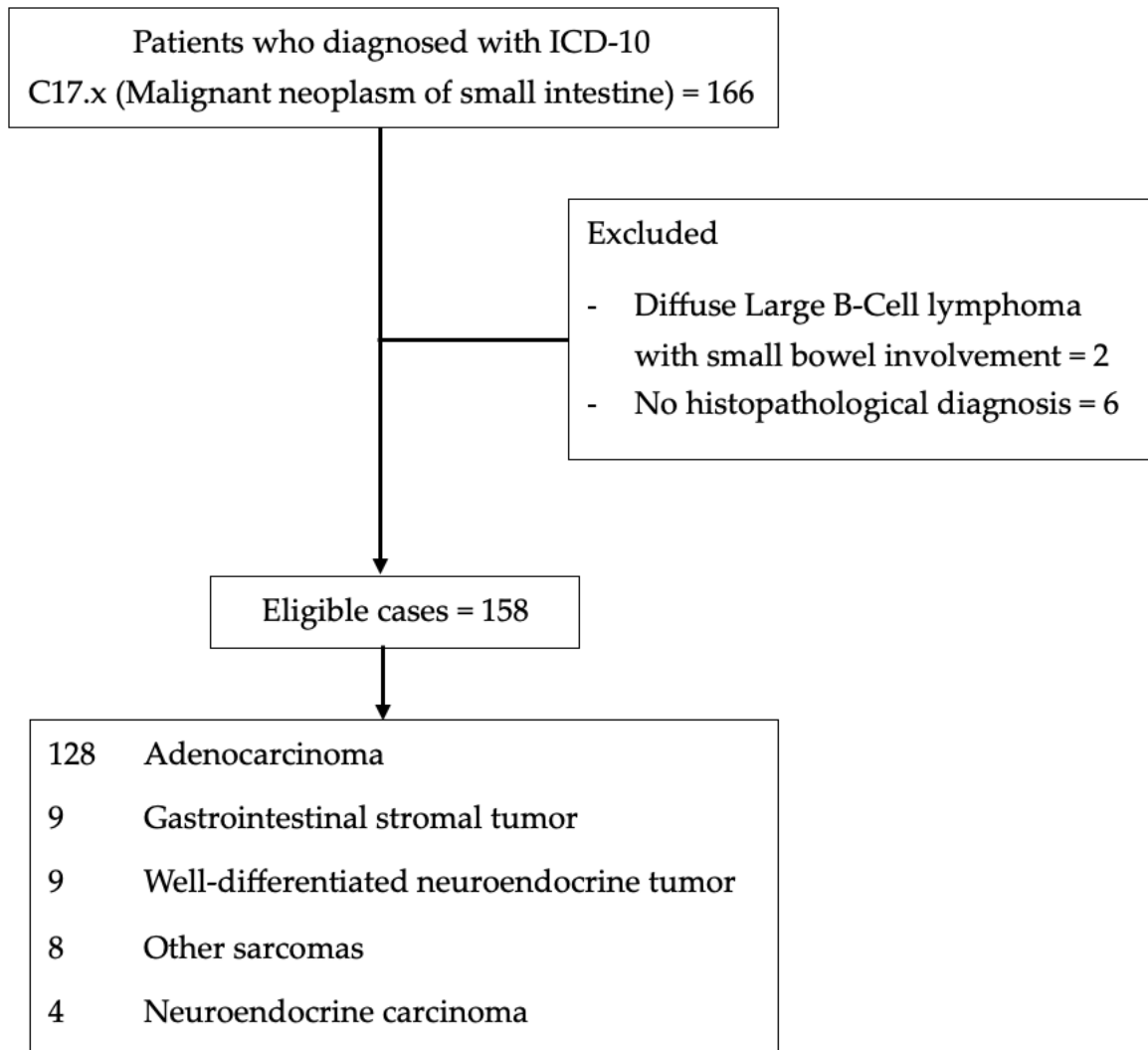

**Figure S1** STROBE flow diagram

Supplement: Supplementary file 1 [file jcm-14-06962-s001.zip › jcm-3846468-supplementary.pdf]
